# The Saddle Projection Graft

**DOI:** 10.1093/asjof/ojae071

**Published:** 2024-08-30

**Authors:** Michael E Nissan, Jon Kurkjian

## Abstract

This paper and the accompanying video present a novel technique developed by the senior author to reliably control nasal tip projection and rotation with a midline cartilage graft that is nonbulky; easily reproducible; and conceptually intuitive. The saddle projection graft is a cartilage graft ∼5 by 15 mm in size fashioned from the nasal septum. A 15 blade is used to create a sagittal groove in the cartilage, which is then inset onto the anterior septal angle with the septum fitting within the sagittal groove. It is positioned to project anteriorly to the position of the desired nasal tip and the graft is inset with sutures. The tip is then unified upon this structurally stable unit. The midline position helps avoid the introduction of new deviation or asymmetry of the nasal tip. The cartilage dimensions do not demand a large stock of cartilage. The technique does not cause external valve obstruction or visibility. Most notably, the saddled position of the graft benefits from the direct support of the sturdy septal cartilage as it opposes the forces of scar maturation, resulting in predictable control of nasal tip projection and rotation.

**Level of Evidence: 5 (Therapeutic):**

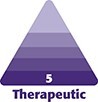

This paper and the accompanying video present a novel technique developed by the senior author to reliably control nasal tip projection and rotation with a midline cartilage graft that is nonbulky, easily reproducible, and conceptually intuitive (Video).

Control of nasal tip projection is a key element of rhinoplasty. The loss of tip projection can have a detrimental impact on the result, as the contour of the entire dorsum depends on a tip that projects beyond the rest of the dorsum, thus creating a desirable supratip break and providing definition to the lower third of the nose. The impetus for the creation of the saddle projection graft was the senior author's disappointment with both septal extension grafts and columellar strut grafts, both independently and used in combination.

This study was not submitted for approval to an institutional review board. It is based on a case series performed by the senior author that was done to maximize patient results without the intention of publishing the technique. It hews to the basic principles of the Declaration of Helsinki. Patient self-determination and the right to make informed decisions were ensured through the informed consent process that each patient underwent prior to surgery. All surgeries were performed for the purpose of maximizing patient safety and satisfaction with surgery, and not for the purpose of this study, which only occurred retrospectively when it was realized that the technique produced favorable results.

The saddle projection graft is fashioned from a portion of the septum that is sturdy, at least 2 mm thick, and without deviation. Typically, the cephalic edge of the harvested septal cartilage near the osseocartilaginous junction has adequate thickness.^1^ The saddle projection graft is relatively small—we typically harvest a piece no larger than 5 by 15 mm in size (Figure 1). Next, a 15 blade is used to create a sagittal groove in the cartilage (Figure 2). The cartilage is then inset onto the anterior septal angle with the septum fitting within the sagittal groove. It is positioned to project anteriorly to the position of the desired nasal tip. A 25-gauge needle can be used to hold the graft in place. The graft is inset with a through-and-through 5-0 polydioxanone mattress suture. Additional simple sutures are placed on the cephalic and caudal edges of the graft to provide rotational stability (Figures 3, 4). Nasal projection is then fine-tuned by trimming the anterior tip of the graft with scissors. Next, the tip is unified upon this structurally stable unit per the preference of the operating surgeon (Figures 5, 6). A schematic representation of the saddle projection graft is also shown (Figure 7). Preoperative (Figure 8) and 7-month postoperative (Figure 9) photographs are included for a patient who underwent rhinoplasty with saddle projection graft. The photographs demonstrate a refined, well-supported tip with increased projection and rotation and without prominence in the nasal vestibule on worm's eye view. An additional case is shown for a patient with a different preoperative morphology who also underwent rhinoplasty with septal projection graft (Figures 10, 11).

**Figure 1.**
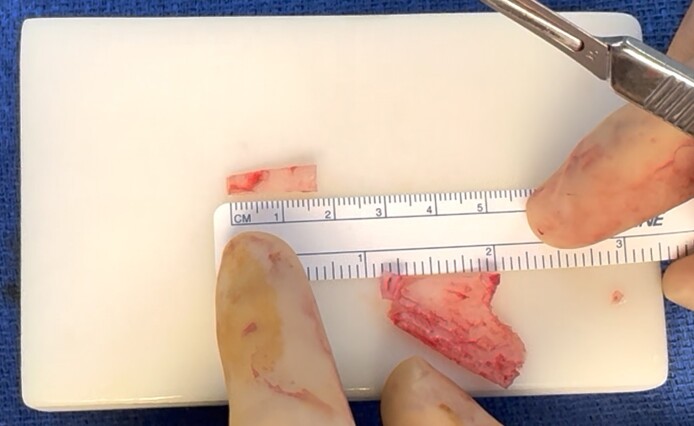
The saddle projection graft (center) harvested from the septal cartilage (lower right).

**Figure 2.**
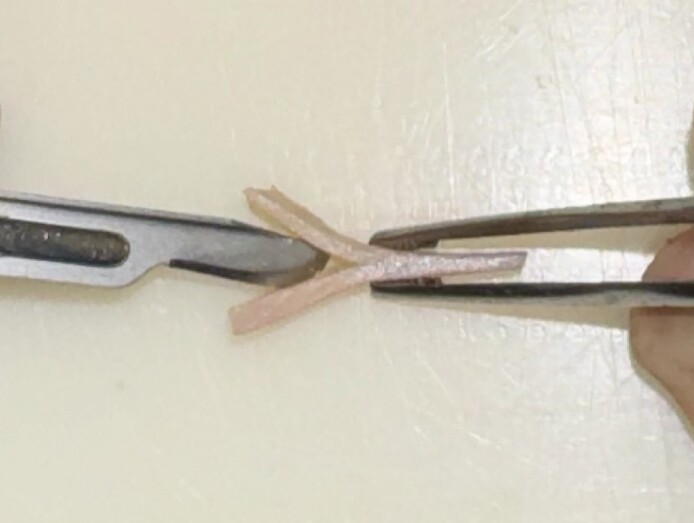
Anteroposterior view of the saddle projection graft, demonstrating the sagittal groove.

**Figure 3.**
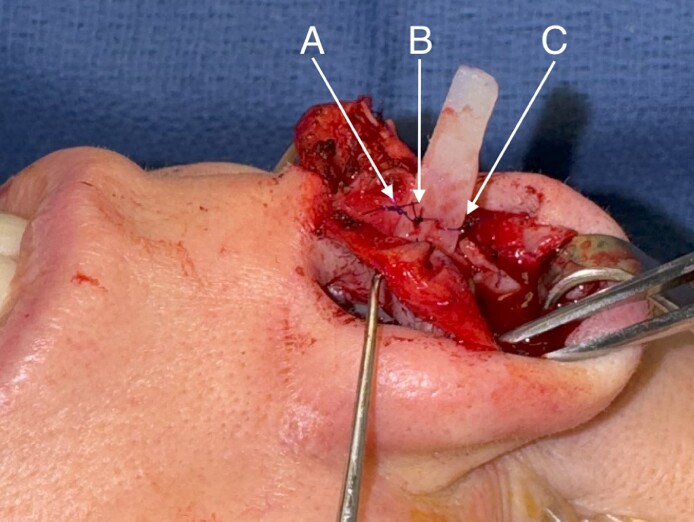
Saddle projection graft inset with caudal simple suture (A), mattress suture (B), and cephalic simple suture (C). Intraoperative photograph of a 27-year-old female.

**Figure 4.**
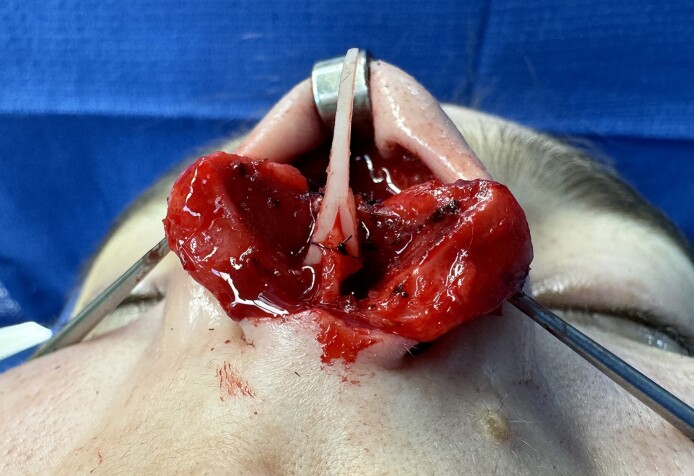
Basal view of the saddle projection graft status post inset, demonstrating the anterior septal angle fitting within the sagittal groove. Intraoperative photograph of a 27-year-old female.

**Figure 5.**
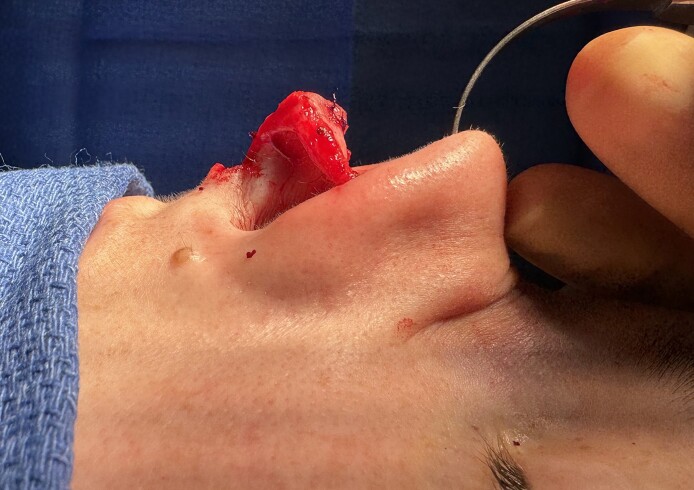
Lateral view of the saddle projection graft status posttip unification. Intraoperative photograph of a 27-year-old female.

**Figure 6.**
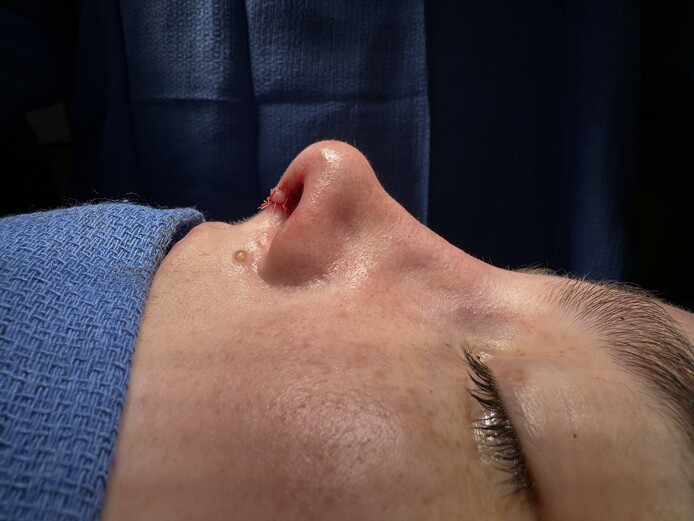
Immediate postoperative photograph of a 27-year-old female.

**Figure 7.**
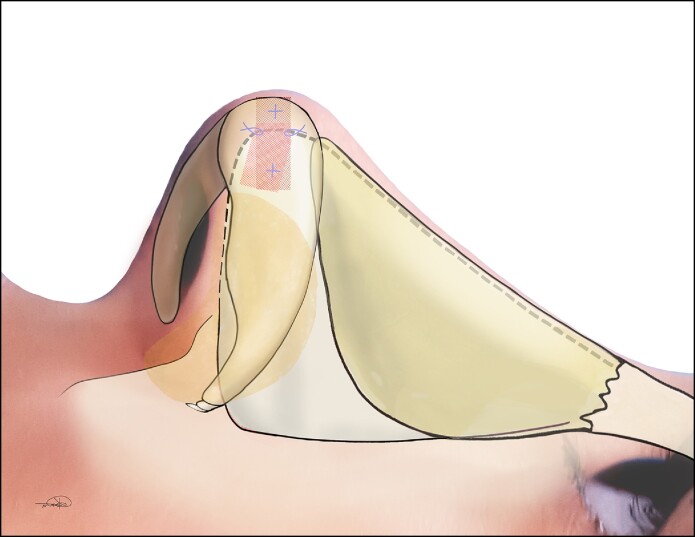
Schematic representation of the saddle projection graft. Illustration by Kate Mackley.

**Figure 8.**
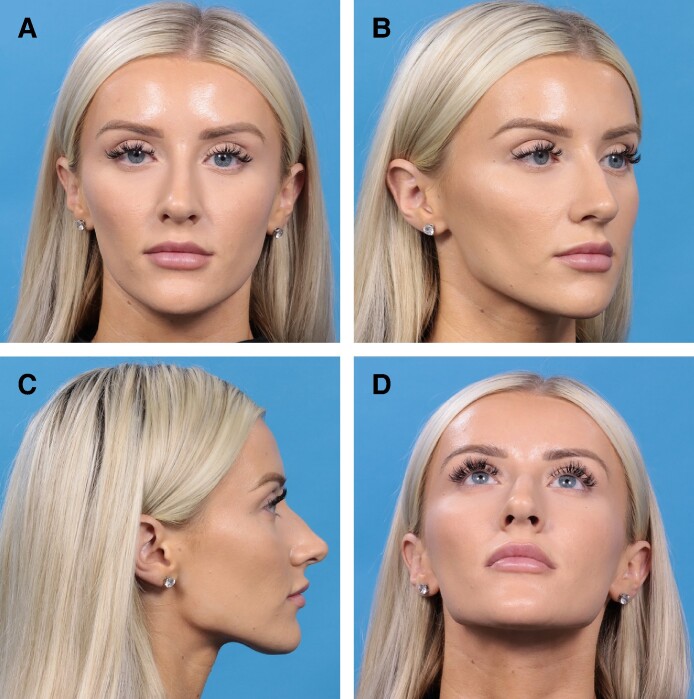
A 26-year-old female, preoperative photographs. (A) Anteroposterior view; (B) oblique view; (C) lateral view; (D) basal view.

**Figure 9.**
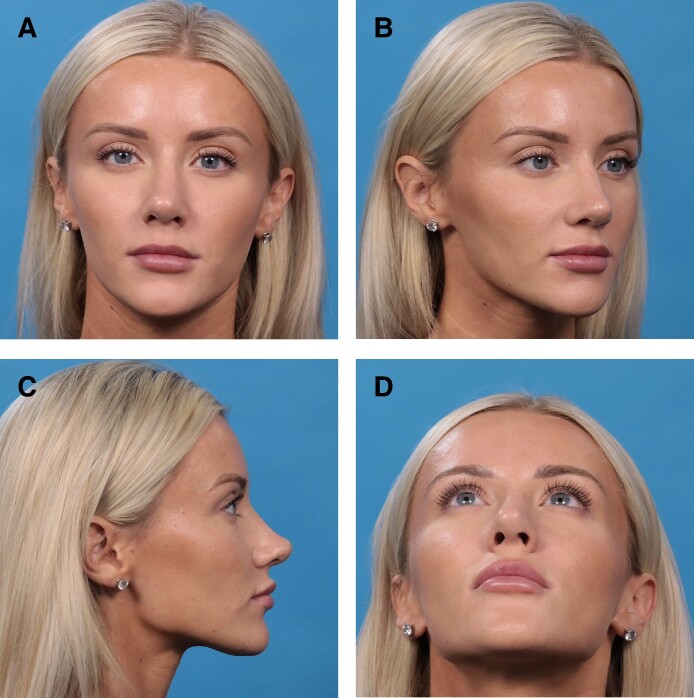
A 26-year-old female 8 months status postrhinoplasty with saddle projection graft. (A) Anteroposterior view; (B) oblique view; (C) lateral view; (D) basal view.

**Figure 10.**
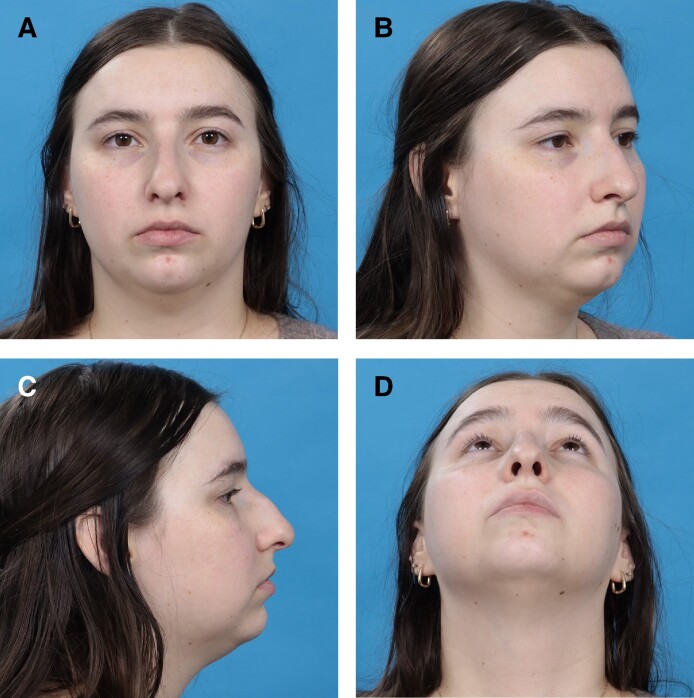
A 27-year-old female, preoperative photographs. (A) Anteroposterior view; (B) oblique view; (C) lateral view; (D) basal view.

**Figure 11.**
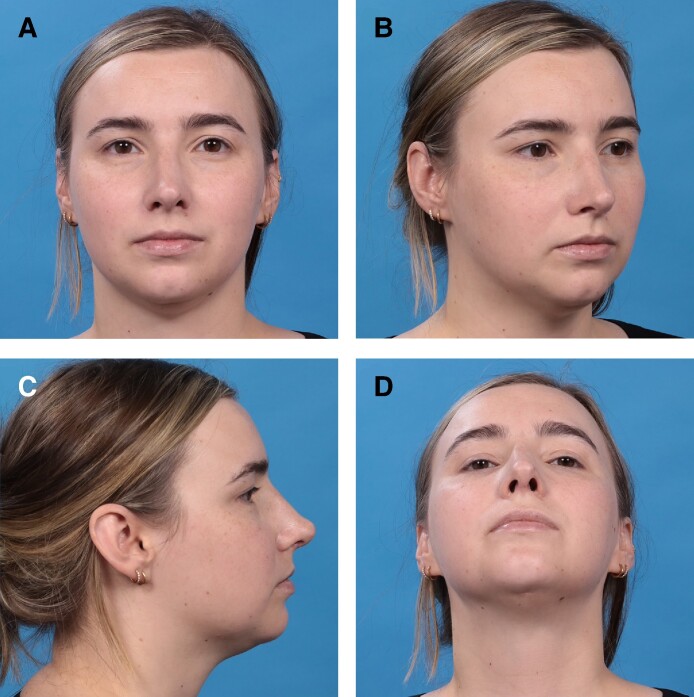
A 27-year-old female 6 months status postrhinoplasty with saddle projection graft; suction lipectomy of neck; chin implant; and malar fat grafting. (A) Anteroposterior view; (B) oblique view; (C) lateral view; (D) basal view.

## DISCUSSION

Control of the nasal tip position has always been a challenge for rhinoplasty surgeons. The initial description of the septal extension graft by Byrd et al in 1997 was a major step forward in rhinoplasty.^2^ Many variations have been described in the literature, all with advantages and drawbacks.

The initial description of the septal extension graft included 3 main types which are still used today by rhinoplasty surgeons around the world.

The batten-type septal extension graft has the advantage of requiring minimal cartilage. Unfortunately, because of its inset, the graft often results in bulkiness in the external nasal valve and visibility on worm's eye view. Additionally, its inset on one side of the septum introduces inherent structural weakness and visual imbalance.

Spreader-type septal extension grafts, in contrast, produce a very stable construct—however, they require very long segments of cartilage usually only available from costal cartilage. They are also not midline and can potentially contribute to dorsal width. Increasingly, spreader grafts are not required with adoption of midvault cartilage preservation techniques.

The direct caudal septal extension graft has the advantage of being a midline structure, but it is structurally weak due to a lack of cartilage overlap. It also has the disadvantage of often requiring a large piece of cartilage.

Alternatively, a columellar strut is employed by many surgeons for control of tip projection. However, it has been shown that this graft does not necessarily increase tip projection.^3,4^ Without costal cartilage, there is rarely enough material to construct a columellar strut that reaches from the columellar soft tissue pocket to the desired tip position. This is exacerbated by the fact that the tip must be overprojected when using only a columellar strut in order to compensate for the unpredictable settling of soft tissue.

The saddle projection graft is a novel technique that offers reliable control of nasal tip projection, rotation, and supratip break. The midline position helps avoid the introduction of new deviation or asymmetry of the nasal tip. The cartilage dimensions do not demand a large stock of cartilage, and thus may be of particular benefit in secondary cases where septal cartilage is often limited. The minimal bilateral overlapping cartilage segments are positioned anteriorly enough to avoid external valve obstruction and visibility. Most notably, the saddled position of the graft benefits from the direct support of the sturdy septal cartilage, as it opposes the forces of scar maturation. The strength of the graft is derived from the solid cartilage at the apex of the split interacting directly with the anterior edge of the septum. The strength of the septal cartilage is thus transmitted to the graft through this interface. The split portions serve to secure the graft to the septum to ensure that it does not tilt. The sutures on the cephalic and caudal edge protect against unwanted rotation. This orientation of the graft on top of the septal cartilage results in predictable control of nasal tip projection and rotation.

It is important to note the potential complications and limitations with the saddle projection graft. When the nasal septum is not sufficiently thick (at least 2 mm), then the cartilage will not withstand a sagittal split without fracturing. For thin cartilage, we recommend using a traditional septal extension graft. Albeit, in our experience, it is rare to encounter septal cartilage that is not 2 mm thick along the cephalic portion of the septum. Additionally, when the nasal septum cannot be set in the midline as is the case for a severely traumatized nose or significantly asymmetric facial skeleton, then the saddle projection graft is not necessarily recommended, as the patient will benefit from a traditional septal extension graft placed to one side of the deviated septum. This technique, of course, assumes the curvature of the traditional septal extension graft is matched perfectly for the degree of septal deviation. We have also found that the saddle projection graft results in minimal “settling” of the nasal tip, which is a phenomenon often seen with septal extension grafts and columellar struts where there is a subtle loss of projection and rotation during the healing process. For this reason, we caution surgeons against the common practice of constructing a slightly overprojected and rotated nose in an effort to compensate for the predictably unpredictable nasal tip settling. These accommodations will likely persist throughout the healing process and affect the final result. Additionally, the septal projection graft results in tip stiffness similar to a traditional septal extension graft. For both the septal projection graft and a traditional septal extension graft, we counsel patients on this preoperatively and explain that the predictability of the result comes at the cost of nasal tip flexibility.

In the senior author's practice, 18 patients have undergone rhinoplasty with saddle projection graft. They range in age from 17 to 58 years. Seventeen cases were primary rhinoplasty and 1 case was a secondary rhinoplasty. All patients were females. The technique has been employed by the senior author for 11 months at the time of this publication. Nasal tip projection and rotation have been reliably maintained in all patients who have undergone rhinoplasty with the use of the saddle projection graft. We attribute the success of the technique to its inherent design, as the cartilage-on-cartilage construct does not rely on the strength or longevity of the sutures, or the unpredictability of a maturing scar. Regardless, further studies should be done to quantitatively verify the technique in terms of nasal tip projection and rotation.
